# Quantitative Kernel estimation from traffic signs using slanted edge spatial frequency response as a sharpness metric

**DOI:** 10.1038/s41598-026-40556-w

**Published:** 2026-02-19

**Authors:** Amit Pandey, Mohd. Zubair Akhtar, Nandana Kappuva Veettil, Bernhard Wunderle, Gordon Elger

**Affiliations:** 1 University of Applied Sciences, Institute of Innovative Mobility (IIMo), Research group Sensor Technology and Data Fusion for Environmental Perception, Esplanade 10, Ingolstadt 85049 Germany; 2 Chemnitz University of Technology, Reichenhainer Str. 70, Chemnitz, 09126 Germany

**Keywords:** Automotive camera, Sharpness, E-SFR, State monitoring, Kernel, Blurring, Applied optics, Imaging and sensing

## Abstract

Sharpness is a critical optical property of automotive cameras, measured by the spatial frequency response (SFR) within the end-of-line (EOL) test after manufacturing. This work presents a method to estimate the blurring kernel of an automotive camera, which could be the first step toward state monitoring of automotive cameras. To achieve this, Principal Component Analysis (PCA) was performed, using synthetic kernels generated by Zemax. The PCA model was built with approximately 1300 base kernels representing spatially variant point spread functions (PSFs). This model generates kernel samples during the estimation process. Synthetic images were created by convolving the synthetic kernels with reference traffic sign images and compared with real-life data captured by an automotive camera. These synthetic data were utilized for algorithm development, and later on, validation was performed on real-life data. The algorithm extracts two $45\times 45$ pixels regions of interest (ROIs) containing slanted edges from the blurred image and crops matching ROIs from a reference sharp image. Each candidate kernel was used to blur the reference ROIs, and the resulting SFR was compared with the blurred ROIs’ SFR. Differential evolution optimization minimizes the SFR difference, selecting the kernel that best matches the observed blur. The final kernel was evaluated against the true kernel for accuracy. The structural similarity index measure (SSIM) between the original and estimated blurred ROIs ranges from 0.808 to 0.945. For true vs. estimated kernels, SSIM varies from 0.92 to 0.98. Pearson correlation coefficients range from 0.84 to 0.99, Cosine similarity from 0.86 to 0.99, and mean squared error (MSE) from $1.1 \times {10}^{-5}$ to $8.3 \times {10}^{-5}$. Validation on real-life camera images showed that the SSIM between the estimated and blurred ROI was >0.82, showing promising accuracy in kernel estimation, which could be used towards in-field monitoring of camera sharpness degradation.

## Introduction

Advanced driver assistance systems (ADAS)^1,2^ and autonomous vehicle technologies are increasingly being introduced on one hand to support the driver and on the other hand to enable greater autonomy. In ADAS, cameras play a crucial role in object detection, collision avoidance, lane departure warnings, and traffic sign recognition due to their ability to interpret text and color. Cameras improve safety by providing critical situational and environmental awareness. High image quality, assessed by spatial frequency response (SFR) measurements, is essential for reliable performance in diverse driving environments. However, camera images are often blurred because of factors such as optical aberrations, sensor characteristics, and motion blur. The objective of the camera is assembled during production with an accuracy of less than 5 $\upmu \textrm{m}$. This is done because of the strict sharpness requirements for sensors used for safety-critical features. To ensure that the tolerance criteria are met, end-of-line (EOL) tests are performed where the sharpness of each camera is measured to assess whether it meets the required specifications and can be classified as pass or fail. The SFR, defined in the ISO standard^3^, is the metric that measures the sharpness in the EOL tests. Due to thermal expansion during operation, the sharpness of a camera changes. In addition, aging effects, such as shrinkage of plastic materials, influence sharpness. Image sharpness is an important factor that influences the accuracy of machine vision algorithms. Although its exact impact on advanced object detection models such as YOLO^4^ is unclear, sharpness becomes critical when detecting distant or small objects^5^, as it allows for a clear detail resolution for successful detection. In contrast, blurred images can result in misinterpretation or in a failure to detect important features such as traffic signs or pedestrians. Therefore, ensuring that automotive camera systems consistently produce sharp images is vital for safety-critical applications.

A widespread method to quantify image sharpness is the slanted edge modulation transfer function (MTF)^6,7^. This method involves analyzing a high-contrast edge, slightly tilted relative to the sensor pixel array. The procedure for extracting the edge spread function (ESF), deriving the line spread function (LSF), and computing the MTF is detailed in^8^. According to ISO 12233^3^, the sharpness parameter is named the spatial frequency response (SFR). It is calculated using tools such as SFRmat^9^, an open-source MATLAB program. In this work, the SFR was calculated using an algorithm inspired by the Python program available on GitHub by Ufuk Onder^10^. Another measure of optical performance is the point spread function (PSF), which captures how the system images a point source. PSF characterizes the spread and nature of aberrations, offering a quantitative measure of optical quality. From an optical systems perspective, the PSF is determined jointly by the lens design (e.g., element curvatures, spacing, aperture, and coatings), the focus setting and field angle, and the sensor characteristics. The spatially variant PSF is hence different for different cameras.

In machine vision, the blurring kernel is often referred to interchangeably with the PSF^11,12^. The image formation process is modeled as a convolution of a high-resolution image $\textbf{S}$ by a kernel $\textbf{K}$, with the addition of white noise ($\boldsymbol{\eta }$), as shown in equation 1^13^. Like the PSF, the kernel describes how light from a point source spreads as it passes through the optical system and forms the image on the sensor. In this work, the kernel was treated as a down-sampled version of the PSF, which was a computationally efficient representation suitable for image restoration and de-blurring tasks.

1 $$\begin{aligned} \textbf{I} = \textbf{S} \circledast \textbf{K} + \boldsymbol{\eta } \end{aligned}$$

**Fig. 1:**
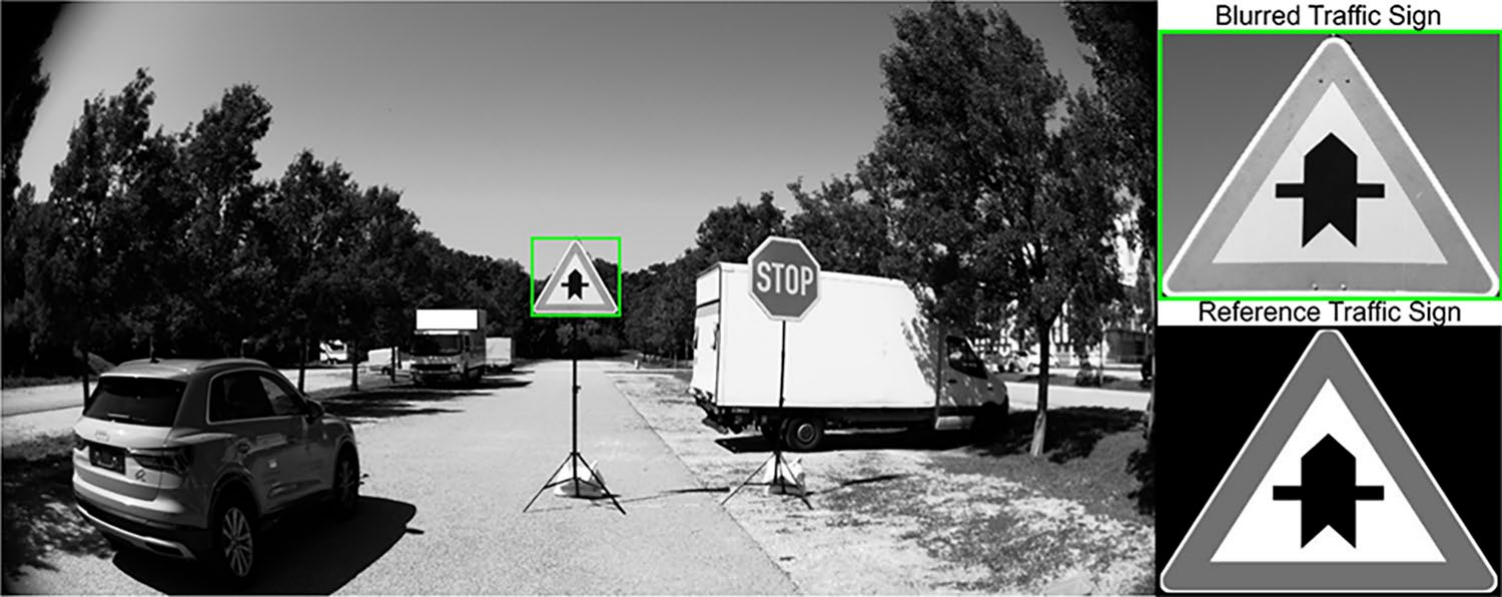
A recreated driving scenario for data collection, including traffic signs for slanted edge SFR calculations. For each static scene, the modified automotive camera was moved $\pm 30$ $\upmu \textrm{m}$ and the same scene was captured. A reference image of a similar traffic sign is also shown for comparison.

One application of kernel estimation is in the field of predictive maintenance and state monitoring of automotive cameras. The cameras are exposed to various stressors, including vibrations, temperature fluctuations, and mechanical wear. Over time, these factors contribute to a gradual degradation in optical performance^14^ and, hence, degradation of data-driven ADAS functions. Estimation of the spatially variant blurring kernel or PSF provides a way to assess the current state of the camera system. State monitoring can be useful in predicting the remaining useful life of the sensor, allowing proactive maintenance or timely sensor replacement, ensuring that the critical imaging components of the vehicle remain reliable (Fig. 1).

Estimating a kernel from an image is ill-posed, as the convolution process results in a loss of information. Many combinations of sharp images and kernels can produce the same blurred result^11^. Thus, kernel estimation requires prior knowledge of either the sharp image or the kernel. An image restoration process that first estimates the kernel and then deconvolves the image is called blind deconvolution. If the kernel is known for image restoration, the process is called non-blind deconvolution, both of which remain challenging due to high-frequency loss and sensor noise^15^. Jizhou Li et al.^16^ proposed calibration-free 3D PSF estimation for fluorescence microscopy using Kirchhoff-based basis functions and optimization under Poisson-Gaussian noise. A. Lau et al.^17^ introduced a two-step blind deconvolution for adaptive optics imaging, improving robustness with analytical PSF models and object priors. Aftab Khan et al.^18^ worked with genetic algorithms and BRISQUE for blind deblurring from camera shake, iteratively refining the PSF estimates to recover complex motion blur without prior assumptions. With deep learning, convolutional neural networks (CNNs) trained on paired blurred-sharp images, such as those shown by Nah et al.^19^, learn to invert blur by implicitly modeling kernels. Hybrid approaches, like Kupyn et al.^20^, combine CNNs with physical priors for improved balance. However, such models require large datasets that are difficult to acquire in real-world automotive scenes, and their computational demands limit use in embedded hardware. In addition to these classical and learning-based approaches, several other blind deblurring methods are directly relevant as comparison baselines for this work. Pan et al.^21^ (dark-channel prior) formulate blind deblurring as a joint optimization over a sharp image and a motion kernel, using the dark channel prior to regularize natural images and achieve strong performance on complex motion blur. More recent methods, such as cross-scale self-supervised deblurring via implicit neural representations by T. Zhang et al.^22^ and blind deconvolution with a generative-based kernel prior and initializer via latent encoding by J. Zhang et al.^23^, further strengthen the image and kernel priors by learning them from data. These approaches are primarily designed to maximize perceptual restoration quality on general natural images, and the estimated kernels are treated as internal latent variables rather than explicit, sensor-specific PSFs. Also, most of the current works in blind image deconvolution are developed for motion or shake blur. In contrast, the present work uses a physically motivated PSF dataset generated from an automotive lens model, meant for localized kernel estimation due to environmental stressors or age. This method reduces the PSF dataset to a low-dimensional kernel manifold using PCA, and drives kernel estimation with a slanted-edge SFR cost tailored to traffic sign imagery. However, the methods of Pan et al.^21^ and the recent INR- and generative-prior-based methods^22,23^ are used as baselines to benchmark kernel estimation accuracy on traffic sign images.

Recent literature on sharpness evaluation, including the survey by Zhu et al.^24^, classifies sharpness evaluation methods into four major categories: spatial domain-based, spectral domain-based, learning-based, and combination methods. Most no-reference (NR) sharpness evaluation techniques rely on gradient-based operators (e.g., Sobel^25^, Prewitt^26^), local variance^27^, wavelet energy^28^, or features extracted using CNNs^29^ and regression models^30^. While these methods provide global or local sharpness scores, they do not directly estimate the underlying blur kernel or PSF responsible for image degradation.

**Fig. 2:**
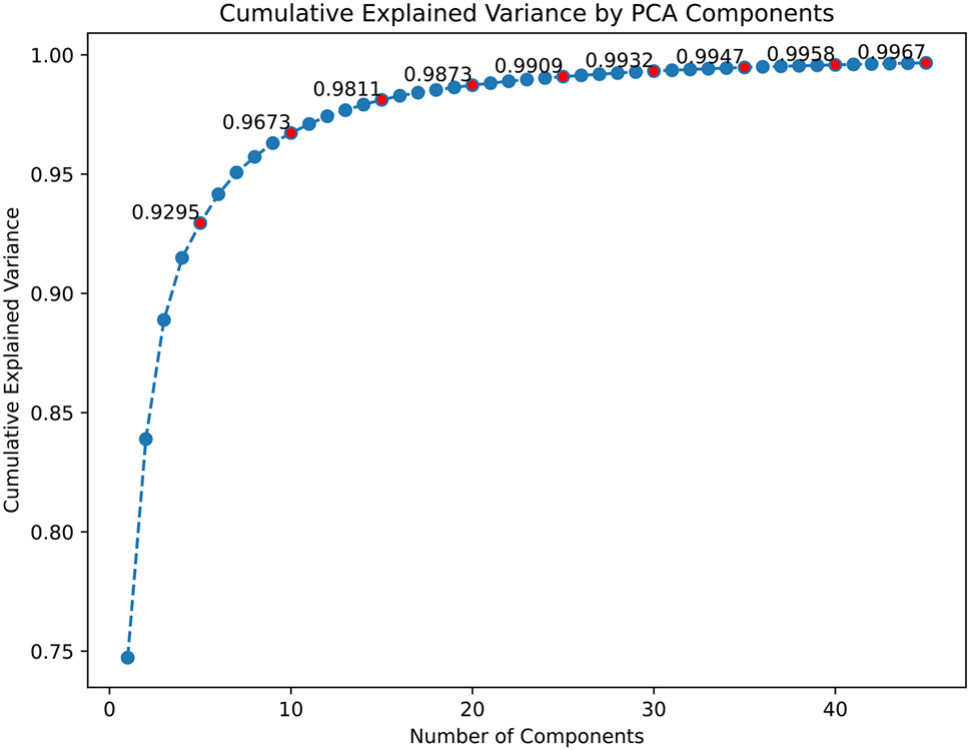
Cumulative explained variance as a function of the number of PCA components, showing that over 99 percent of the variance was captured within the first 25 components. The data was labeled for every fifth point on the x-axis and also highlighted in red.

Unlike prior approaches that return scalar sharpness scores, the method proposed in this work explicitly estimates a spatially variant 2D blur kernel based on slanted edge SFR matching. It operates in a full-reference setup, leveraging both a sharp template image and a blurred observation similar to classical full-reference quality metrics, but tailored for PSF estimation. In doing so, it addresses a critical limitation in NR sharpness metrics: the lack of interpretability and traceability to the optical root cause of blur. Moreover, the method in this work was informed by optical design knowledge, using PCA-reduced kernels derived from Zemax simulations as a physical prior, which was absent in learning-based NR methods. While deep learning-based techniques such as DeepBIQ^31^ achieve strong correlation scores (e.g., Pearson correlation coefficient > 0.95 on benchmark datasets^24^), they lack applicability in embedded or in-field diagnostic settings where sharp references or field angle priors may be available (Fig. 2).

This work introduces a method for estimating blur kernels from traffic sign images. As the method relies on slanted edges within the signs, the resulting kernel refers to a specific field angle, that is, the location of the edge in the image. Since blur kernels vary spatially, one kernel cannot “de-blur” or deconvolve the entire image, but only a local region near the slanted edge. The goal was to estimate the optical blur kernel of an automotive camera to assess the condition of the system, using prior knowledge of the optical design of the camera. Thus, the proposed method complements existing sharpness metrics by bridging the gap between image-based sharpness assessment and optical kernel estimation, offering a physics-informed, ROI-based diagnostic tool for automotive camera health monitoring.

Dimensionality reduction methods such as PCA^32–34^ help analyze the PSF data by transforming correlated variables into uncorrelated principal components. In the context of kernel estimation, PCA is performed to reduce the complexity of the data by isolating the dominant modes of variance. This process simplifies subsequent data analysis and makes kernel interpretation simpler by identifying the required dominant components. With the PCA model, a kernel can be recreated. More information on the implementation of the model and its usage is provided in Section “Methodology”.

This work is structured into the following three sections: methodology, results and discussion, and conclusions and outlook. The methodology describes the PCA performed to define a model that generates kernel samples during the estimation process. The section also includes the synthetic data and real-life camera data utilized to develop and validate the algorithm. The section concludes with a detailed description of the algorithm, including mathematical definitions of the SFR calculations and the differential evolution optimizer used to find the best matching kernels. The methodology section is followed by the results and discussion section, in which the results of the algorithm development and testing on the synthetic data were presented and discussed with various correlation metrics. The results of the final algorithm on the validation data were also presented and discussed. The work is concluded in the next section, which briefly defines the findings of this work and puts it in context with real-world scenarios. This section also describes the shortcomings of the method and future work in the field.

## Methodology

**Fig. 3:**
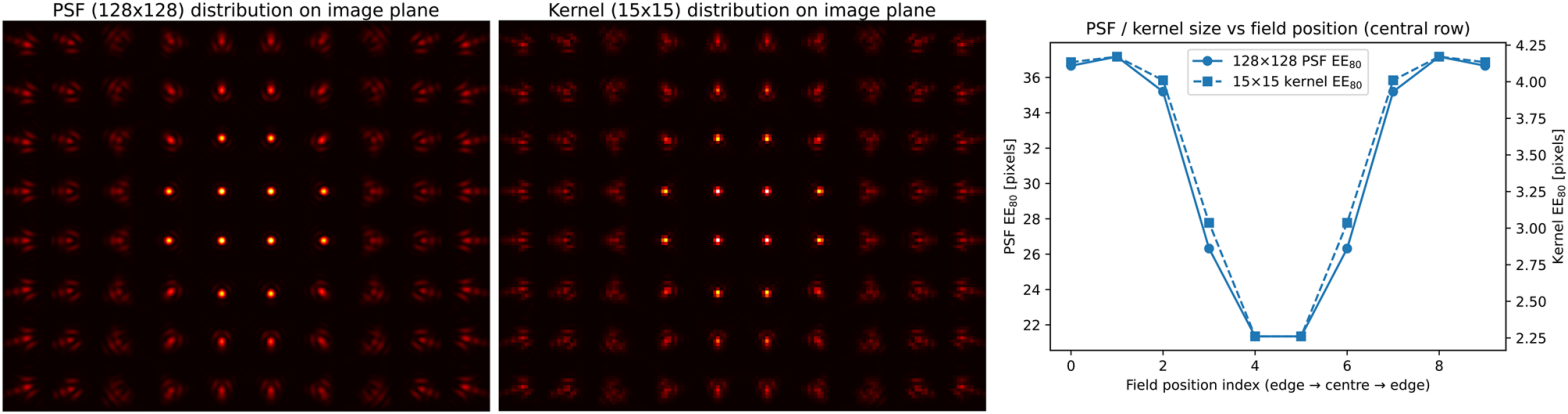
Spatial distribution of the simulated PSFs ($128\times 128$ pixels, left) and the corresponding downsampled blur kernels ($15\times 15$ pixels, center) across the image field. The plot on the right shows the PSF and kernel encircled–energy diameter $\textrm{EE}_{80}$ versus field position (central row), demonstrating that the reduced kernel representation preserves the field-dependent variation in PSF size.

### Kernel and image data

PCA was performed on a dataset of synthetic PSFs to construct a representative basis for generating spatially variant kernels. These synthetic PSFs were simulated using Ansys Zemax for a series production, multi-lens (six-element) automotive camera objective. Each PSF in the dataset corresponds to a unique combination of defocus and object angle, capturing the key optical variations relevant to real-world imaging conditions. The PSF data were extracted as intensity values in a $128 \times 128$ array and then down-sampled to a $15 \times 15$ array through a bicubic interpolation. The downsampled PSF will be termed the “kernel” for the course of this work. The downsampling was done to ensure that image convolution, PCA, and kernel estimation can be performed with reasonable computational resources. The Zemax model, the PSF dataset, and the corresponding down-sampled kernels used in this work were therefore defined for one specific camera configuration. Kernels produced by a different camera, even if qualitatively similar, will in general lie outside this manifold because the underlying balance of aberrations and spatial sampling on the sensor was different. In this data, the spatial variance of the PSF was quantified by calculating the encircled-energy (EE) radius $r_{80}$ for each simulated PSF on the field grid. For a given PSF, normalized to unit energy, the radius around the PSF centroid that contains 80% of the total intensity ($\textrm{EE}_{80}$) is calculated. Figure 3 shows $r_{80}$ as a function of field position along a center-to-edge line for one representative defocus plane. The PSF size increases monotonically from the image center to the outer field positions, demonstrating spatial variation of the blur across the image plane. Together with the PSF and the kernel mosaics, Fig. 3 illustrates that the PCA kernel model was trained on a spatially variant PSF manifold.

In the next step, ten blurred images were created by convolving ten different test kernels with a sharp cross-roads traffic sign image. The convolution process produces output images of the sharp traffic sign blurred to different extents based on the convolution kernel. This convolved image will be referred to as the ”blurred” image in the course of this work, and the sharp image will be referred to as the “reference” image. For the validation of the algorithm, real-life images were captured using an automotive camera. This automotive camera has the same objective for which the Zemax model was used to generate the PSFs. The captured images contain the same traffic sign as that in the synthetic data for comparison. For the data collection process, a state-of-the-art automotive camera was modified by separating the objective and the sensor package^5^ (which are assembled together with a thermosetting adhesive to prevent relative motion). The camera was modified in such a way that moving the sensor relative to the objective was possible, causing defocus. The defocus in this setup was quantified by measuring the relative displacement with a micrometer gauge. Traffic sign images were captured at various defocus intervals ($\pm 30$ $\upmu \textrm{m}$), and validation of the algorithm developed was performed on these real-life images.

### Principal component analysis

Estimating the PSF of an optical system is a mathematically complex task. The Zernike polynomials provide a basis for modeling multiple aberrations up to a very high order, ensuring the inclusion of all the factors required to model the PSF. However, as explained by the authors in the article^35^, the Zernike method for PSF modeling works best for diffraction-limited systems where aberrations are small with high-quality and precise optics. However, in systems like automotive cameras, aberrations dominate the optical behaviour, where the focus is on cost and size reduction. This makes Zernike modeling of the PSF a very complex problem requiring about 100 terms to be able to create a PSF model. This is computationally expensive and would require unrealistic resources. The authors in this work^35^ also discuss how currently there are no numerical models that can account for the mass-produced lens tolerances and be able to model a highly non-linear spatially variant PSF. To overcome this hurdle, this work proposes parameterizing the space of blur kernels using PCA^32–34^, a statistical technique that reduces the dimensionality of correlated variables into a set of uncorrelated principal components that capture maximal variance in the data.

Each $15 \times 15$ kernel was first reshaped to a vector in $\mathbb {R}^{225}$. Given a dataset of $N$ blur kernels, PCA begins by computing the mean kernel $\boldsymbol{\mu } = \frac{1}{N} \sum _{i=1}^N k_i$ and forming the centered data matrix $X = [k_1 - \boldsymbol{\mu }, \dots , k_N - \boldsymbol{\mu }]^T$. The covariance matrix is given by $C = \frac{1}{N-1} X^T X$, and eigenvalue decomposition $C = V \Lambda V^T$ yields eigenvectors $\textbf{v}_j$ and eigenvalues $\lambda _j$, sorted in descending order. Each kernel was approximated as:

2 $$\begin{aligned} \textbf{k} \approx \boldsymbol{\mu } + \sum _{j=1}^{35} \alpha _j \textbf{v}_j, \quad \text {where } \alpha _j = (k - \boldsymbol{\mu })^T \textbf{v}_j \end{aligned}$$

3 $$\begin{aligned} \frac{\sum _{j=1}^{m} \lambda _j}{\sum _{j=1}^{225} \lambda _j}> 95\% \end{aligned}$$

The mathematical necessity of applying PCA in the application of this work arises from the high dimensionality of the kernel. Each $15 \times 15$ kernel was represented in a 225-dimensional vector. This could lead to an optimization process that was computationally very expensive. To address this problem, the work in this article proposes a data-driven approach that uses a dataset of synthetic kernels, generated by Zemax, for the PCA. This step decomposes each kernel into a linear combination of a mean kernel and a set of orthogonal vectors (representing the principal components). The number of principal components was defined in such a way that it captures the majority of dominant variations in the entire dataset.

**Table 1:** Sensitivity of kernel reconstruction quality and DE optimization time to the number of PCA components.

#PCs	Expl. var.	MSE [$10^{-6}$]	SSIM	Pearson	Cosine	$R^{2}$	Time [s]
20	0.9875	$8.0 \pm 11.0$	$0.921 \pm 0.131$	$0.922 \pm 0.145$	$0.954 \pm 0.079$	$0.871 \pm 0.218$	356
25	0.9910	$6.0 \pm 9.0$	$0.940 \pm 0.110$	$0.938 \pm 0.125$	$0.964 \pm 0.070$	$0.896 \pm 0.194$	452
30	0.9933	$4.0 \pm 8.0$	$0.954 \pm 0.096$	$0.953 \pm 0.105$	$0.972 \pm 0.060$	$0.918 \pm 0.168$	583
35	0.9948	$4.0 \pm 7.0$	$0.964 \pm 0.081$	$0.961 \pm 0.092$	$0.977 \pm 0.053$	$0.932 \pm 0.150$	682
40	0.9959	$3.0 \pm 6.0$	$0.970 \pm 0.070$	$0.967 \pm 0.080$	$0.980 \pm 0.046$	$0.942 \pm 0.133$	810
45	0.9968	$3.0 \pm 5.0$	$0.975 \pm 0.057$	$0.972 \pm 0.068$	$0.983 \pm 0.040$	$0.950 \pm 0.116$	996
50	0.9974	$2.0 \pm 4.0$	$0.979 \pm 0.049$	$0.977 \pm 0.058$	$0.986 \pm 0.035$	$0.957 \pm 0.102$	1315

The number of principal components (35) was selected to ensure that a very high percentage of the explained cumulative variance (equation 3) was preserved while minimizing dimensionality. This is shown in Fig. 2, and the various metrics of kernel reconstruction via inverse PCA for various numbers of principal components are shown in Table 1. It can be seen from the table that as the number of principal components increases from 20 to 25, the cumulative explained variance increases to beyond 99%, which was one of the goals for selecting the number of principal components. Beyond this point, the reconstruction metrics improve only gradually, but the optimization complexity increases because the DE search space scales with the number of PCA coefficients. In particular, Table 1 shows that increasing from 30 to 35 components yields a measurable improvement in reconstruction quality, whereas the improvement from 35 to 40 is marginal, while increasing the computation time per 100 iterations. Hence, 35 was chosen as a compromise between computation time and accuracy in kernel reconstruction. For more compute-constrained settings, fewer components (e.g., 30) can be selected at the cost of a small reduction in reconstruction quality.

**Fig. 4:**
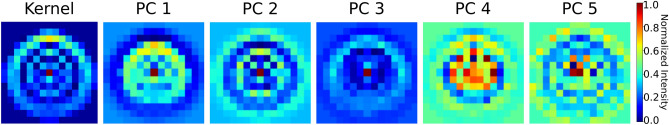
Visualization of one of the original kernels (left) followed by the first five principal components, highlighting the dominant patterns captured by PCA in descending order of explained variance.

An example of the decomposition of a kernel into its first five principal components is shown in Fig. 4. With PCA, the kernel space was reduced to a 35-dimensional representation. A third-degree polynomial regression model was then trained on these coefficients (weights) of the principal component to learn non-linear relationships within the reduced space of the PCs. This polynomial regression model was trained only on the PCA-transformed kernel coefficients and does not incorporate external parameters such as defocus or field angle. Let $\textbf{c} \in \mathbb {R}^{35}$ denote the vector of PCA coefficients for a given PSF. All such coefficient vectors from the Zemax kernel dataset were randomly split into a training set (90%) and a test set (10%). A multivariate polynomial regression model was implemented in the PCA space by generating polynomial features of the input coefficients and fitting a linear regression model in closed form to minimize the MSE between predicted and true coefficients. For polynomial degrees between 2 and 5, the MSE in PCA space on the held-out test set was $3.22\times 10^{-13}$, $5.02\times 10^{-13}$, $7.35\times 10^{-13}$ and $1.81\times 10^{-7}$ for degrees 2, 3, 4 and 5, respectively. Thus, degrees 2–4 yield almost identical, numerically negligible interpolation errors, indicating that the PSF manifold is effectively linear in the PCA space over the sampled region, whereas degree 5 leads to a clear degradation due to overfitting. In our experiments, the regression mapping behaved close to identity mapping for in-distribution coefficients and is therefore optional. It is retained as a lightweight regularization step for potential out-of-distribution coefficient proposals during optimization.

During kernel estimation, a candidate kernel was generated by varying the 35 weights of the polynomial model, which maps the coefficients back to pixel space using the PCA inverse transform, reconstructing a realistic 15$\times$15 kernel. This combined PCA and polynomial regression framework offers a compact representation of the kernel space. Unlike parametric models such as Gaussian- or Zernike-based approximations, it imposes no predefined symmetry or shape constraints and was not as computationally challenging. Instead, it generalizes to complex and asymmetric real-world kernel representations. It is important to note that the model was developed for a specific sensor, i.e., it was designed to work with kernels generated by a known camera. As discussed before, the spatially variant PSF is determined by various factors of the camera system, and these factors vary from camera to camera. Projecting kernels of a different camera system onto the present PCA basis introduces systematic approximation error. This was the main reason why this method is considered to be sensor-specific and why a new PCA basis and regression model would need to be trained from the beginning whenever the optical design or sensor architecture changes. In practice, this involves generating a new PSF dataset, recomputing the PCA basis, and retraining the mapping model for the new system. Performing PCA is not computationally demanding in this workflow. On a workstation equipped with an NVIDIA RTX 4060 Ti (8 GB), the full pipeline executes within seconds. Therefore, when adapting the method to a new sensor, the PCA basis can be recomputed directly, without requiring additional measures such as transfer learning.

The trained model was evaluated by calculating the correlation between a random input kernel and the kernel reconstructed by the model. Correlation was evaluated using the Pearson correlation coefficient, and other metrics, such as the SSIM, Cosine similarity, and MSE, defined in Section "Metrics calculated for estimating goodness of fit", were calculated to evaluate the similarity between the true and reconstructed kernels. The results in Table 1 show that the kernels reconstructed by the PCA model achieve high accuracy.

### Optimization by differential evolution

Optimization of solution and kernel estimation in this work uses differential evolution (DE)^36–38^, which is an algorithm based on the principles of evolution^39,40^. DE is an optimization method that mimics the process of natural evolution using population-based stochastic search. It works by evolving a population of possible solutions over time, using operations such as mutation, crossover, and selection^41^ to explore the search space. DE is useful for problems that are continuous, nonlinear, or high-dimensional, particularly when gradients are hard to compute or do not exist. The benefits of using DE are the balanced approach between exploring new possibilities and fine-tuning promising solutions, which makes it effective for finding global optima in complex high-dimensional scenarios.

In the proposed kernel estimation algorithm, DE optimizes over a 35-dimensional continuous space defined by the principal components of a physically meaningful kernel model. Each candidate in the DE population corresponds to a unique combination of 35 PCA coefficients, representing a distinct kernel hypothesis. In the optimization process, DE generates new candidates through variation of existing parameters by scaling differences between members of the randomly selected population. The crossover operation then mixes the mutated vector with the current candidate, introducing variability, while the selection mechanism ensures that only candidates with lower costs proceed to the next generation. The cost function in the method proposed in this work was defined as the MSE between the estimated and the reference SFR (discussed in more detail in 2.4).

Kernel estimation uses SciPy’s DE optimizer with population size=100, tolerance=$10^{-10}$, mutation=(0.01, 0.99), and recombination=0.95. If DE does not converge, up to four restart attempts are performed using a reinitialized population biased around the best solution found so far, with mutation=(0.05, 1.0) and recombination=0.99.

### Algorithm for Kernel estimation

**Fig. 5:**
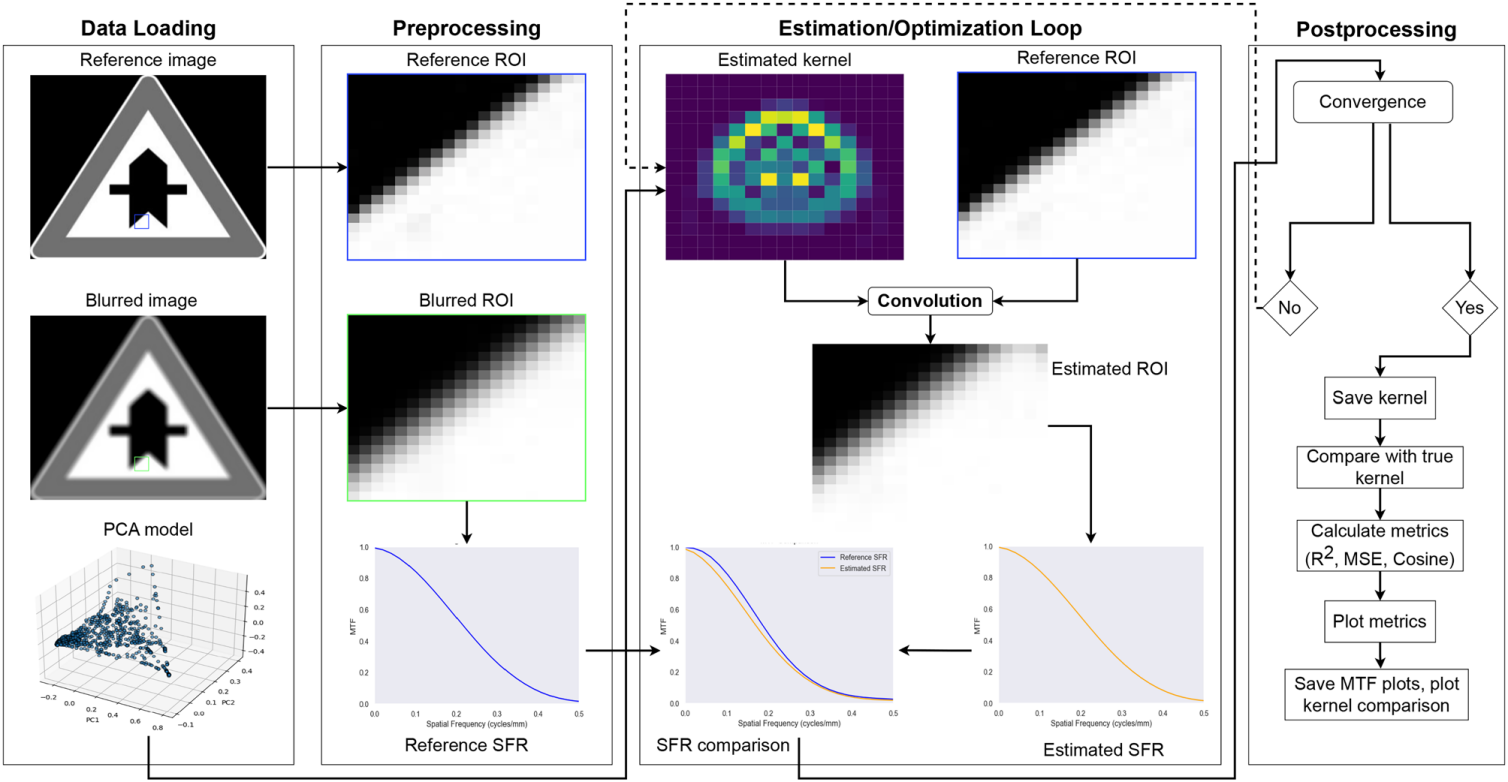
Flow diagram showing the workflow in the algorithm for the kernel estimation process. The loop shows the steps included in the process, such as the ROI extraction from blurred and reference images, and iteratively estimates the blur kernel by comparing the SFR of the convolved reference ROI with the reference SFR. Postprocessing involves evaluating the estimated kernel quality using various metrics and visualizations.

Figure 5 shows the workflow of the kernel estimation algorithm with the intermediate steps. The algorithm begins by loading both the input data and the precomputed PCA model of the kernel space. The input data consists of a sharp reference image and a corresponding blurred image of the same traffic sign. From each image, two regions of interest (ROIs) were extracted, each of size $45\times 45$ pixels. These ROIs contain slanted edges for SFR computation. The algorithm presented in this work used two ROIs because, based on the position of the slanted edge in the image, the SFR will vary. Also, as mentioned by Joshi et al.^11^, there can exist many combinations of kernels and images that will produce the same blurred image after convolution. To avoid bias in the estimation algorithm, a second ROI was introduced. The second ROI also helps during the optimization process by preventing a biased solution from being stuck in a local minimum. In the present camera experiments, shown in Fig. 1, both ROIs were located in the central region of the image. As a result, the validation focused on local kernel estimation at these positions and does not attempt to map PSF variation across the full image plane. Additional ROIs at mid-field and corner positions would be required for full-field characterization. The results presented in Fig. 9 also show that the SFRs of two regions vary slightly from each other under real-life conditions. ROI extraction also has a strong dependence on the distance between the traffic sign and the camera. This was to ensure that the traffic sign was close enough so that an ROI of at least $15\times 15$ pixels can be extracted for the calculation of the SFR on the slanted edge. More ROIs can eventually be added to improve the accuracy of the algorithm at the cost of computation time.

In the next step, a random kernel was sampled from the PCA model, which represents a candidate solution within the kernel space. This candidate kernel was convolved with the reference ROI to generate an estimated version of the blurred ROI. The SFR was then computed for both the blurred ROI and the estimated ROI, and the MSE was calculated between the two SFR curves. The cost function was computed as the sum of the SFR-squared differences over both ROIs, so that the optimizer estimates a kernel that jointly explains both local blur observations.

The DE optimizer iteratively updates the PCA coefficients to minimize this cost. For each new candidate, the process of kernel generation, convolution, SFR estimation, and cost evaluation was repeated. This continues until the best candidate was found or the maximum number of iterations was reached. The candidates were updated based on the mutation and combination parameters defined in the previous section.

The final kernel was compared with the ground truth kernel to estimate the goodness of fit using similarity and correlation metrics defined in Section "Metrics calculated for estimating goodness of fit". Metrics such as the MSE were calculated to evaluate the margin of error between the true kernel and the estimated kernel. After this, kernel visualizations and SFR comparisons were generated, and the results were saved. The optimizer then proceeds to the next blurred image in the dataset, and the process repeats until kernel estimates have been produced for all input blurred images.

Mathematically, given the input parameters $\theta$, the kernel is generated as follows:

1. **Polynomial Transformation:** The parameters $\theta$ are transformed using the pre-trained polynomial model, yielding a representation in the PCA-reduced space.
2. **Inverse PCA Transformation:** The representation from Step 1 is then transformed back to the original kernel space using the inverse PCA transformation, resulting in the 2D kernel, $K(\theta )$.
3. **Convolution:** The estimated kernel, $K(\theta )$, is convolved with the sharp image ROIs, $S_{rois}$, to generate synthetically blurred ROIs, $B_{estimated} = S_{rois} * K(\theta )$ where $*$ is the convolution process, similar to the process defined by equation 1.
4. **SFR Calculation:** The SFR of the originally blurred ROIs, $SFR_{blurred}$, and the estimated blurred ROIs, $SFR_{estimated}$, are calculated.
5. **MSE minimization:** The cost function, $MSE(SFR_{blurred}, SFR_{estimated})$, is minimized with respect to $\theta$ using the DE optimizer.

Although many embedded platforms in automotive systems can support real-time execution of simplified image quality metrics such as SFR, the proposed kernel estimation pipeline is not intended for real-time deployment on embedded hardware. The differential evolution algorithm runs for thousands of iterations to reach an optimized, low-cost kernel estimation based on the number of principal components, which could be computationally intensive for traditional embedded automotive hardware. This pipeline was designed for offline execution on centralized servers, where multiple images can be processed together to estimate degradation trends. Given the slow nature of optical degradation^14^, periodic transmission of a small number of images to original equipment manufacturer (OEM) servers or sensor manufacturers, via secure data sharing frameworks such as GAIA-X^42^, would be a suitable strategy. The estimation algorithm can then run overnight or during maintenance windows and report the camera state back to the vehicle owner.

### Metrics calculated for estimating goodness of fit

To evaluate the similarity between the estimated kernel and the true kernel, we compute several quantitative metrics. The following are the definitions of the metrics calculated:

Pearson Correlation Coefficient: Pearson correlation coefficient^43^ ($\rho$) measures the linear correlation between two variables, in this case, between the estimated and ground truth kernels. For comparison, both the estimated and reference kernels are flattened into 1D vectors before being used to calculate the metric using the equation shown in Equation 4. The values of the Pearson correlation coefficient range from −1 to 1. Values closer to 1 indicate stronger agreement in the spatial structure of the true and estimated kernels. Since the kernels are non-negative, values are typically expected to be in the range 0.8 to 1.0 for a high-quality estimation. Pearson correlation coefficient is defined as:

4 $$\begin{aligned} \rho = \frac{\sum _{i=1}^n (x_i - \bar{x})(y_i - \bar{y})}{\sqrt{\sum _{i=1}^n (x_i - \bar{x})^2} \sqrt{\sum _{i=1}^n (y_i - \bar{y})^2}} \end{aligned}$$

where $x_i$ and $y_i$ are the flattened pixel values of the estimated and true kernels, and $\bar{x}$, $\bar{y}$ are their means.

Cosine Similarity : Cosine similarity^44^ measures the angle between two vectors, providing a scale-invariant measure of their similarity. For kernels, the estimated and ground-truth kernel matrices are flattened and compared. The general values for this metric could lie between −1 and 1. However, since kernels are non-negative arrays, the values of Cosine similarity would lie between 0 and 1, indicating orthogonality (no similarity) or the same direction (perfect similarity), respectively. Cosine similarity is defined as:

5 $$\begin{aligned} \cos (\theta ) = \frac{\sum _{i=1}^n x_i y_i}{\sqrt{\sum _{i=1}^n x_i^2} \sqrt{\sum _{i=1}^n y_i^2}} \end{aligned}$$

Coefficient of Determination ($R^2$) : The $R^2$ score^45^ quantifies how well the estimated values explain the variance in the reference data. For kernels, it is calculated by comparing the flattened estimated and true kernel pixel values. The $R^2$ score could be between -$\infty$ and 1. An $R^2$ score of 1 or close to 1 would indicate that the model is able to estimate kernels that capture all the variance and patterns in the ground truth kernel. $R^2$ score is defined as:

6 $$\begin{aligned} R^2 = 1 - \frac{\sum _{i=1}^n (y_i - x_i)^2}{\sum _{i=1}^n (y_i - \bar{y})^2} \end{aligned}$$

Structural Similarity Index Measure (SSIM) : SSIM^46^ is a perceptual similarity metric that compares two images based on luminance, contrast, and structure. For kernels, it is computed between the 15$\times$15 estimated and reference kernel matrices. For ROIs, SSIM is applied to the slanted edge ROI of the original (blurred) and convolved reference images. The closer the SSIM is to 1 the higher the similarity between the compared kernels or the compared ROIs. SSIM is defined as:

7 $$\begin{aligned} \text {SSIM}(x, y) = \frac{(2\mu _x \mu _y + C_1)(2\sigma _{xy} + C_2)}{(\mu _x^2 + \mu _y^2 + C_1)(\sigma _x^2 + \sigma _y^2 + C_2)} \end{aligned}$$

where $\mu _x, \mu _y$ are the means, $\sigma _x^2, \sigma _y^2$ are variances, and $\sigma _{xy}$ is the covariance of $x$ and $y$. $C_1$ and $C_2$ are stabilization constants.

Mean Squared Error (MSE) : MSE^47^ quantifies the average squared pixel-wise (or frequency-wise) difference between the estimated and true values. For kernels, it is calculated by squaring the difference between each corresponding pixel after flattening. For ROI-based MTF comparisons, it compares MTF values at matched frequencies. The ideal MSE values are very close to zero, indicating that there is a very small error between the original and estimated values. MSE is defined as:

8 $$\begin{aligned} \text {MSE} = \frac{1}{n} \sum _{i=1}^n (x_i - y_i)^2 \end{aligned}$$

## Results and discussion

This section presents the results of the kernel estimation algorithm on synthetically blurred data and real-life automotive camera data, with a comparison and discussion about the difference in the accuracy of the estimation in both cases.

### Kernel estimation on synthetic data

To evaluate the performance of the developed algorithm, 10 kernels were generated within the parameter space of the PCA of the 1300 kernels utilized in the PCA and the polynomial model. These kernels were selected to represent varying degrees of spread of the PSF, hence blur. The reference image shown in Figure 1 was convolved with each kernel, and the resulting images were processed using the kernel estimation algorithm.

**Fig. 6:**
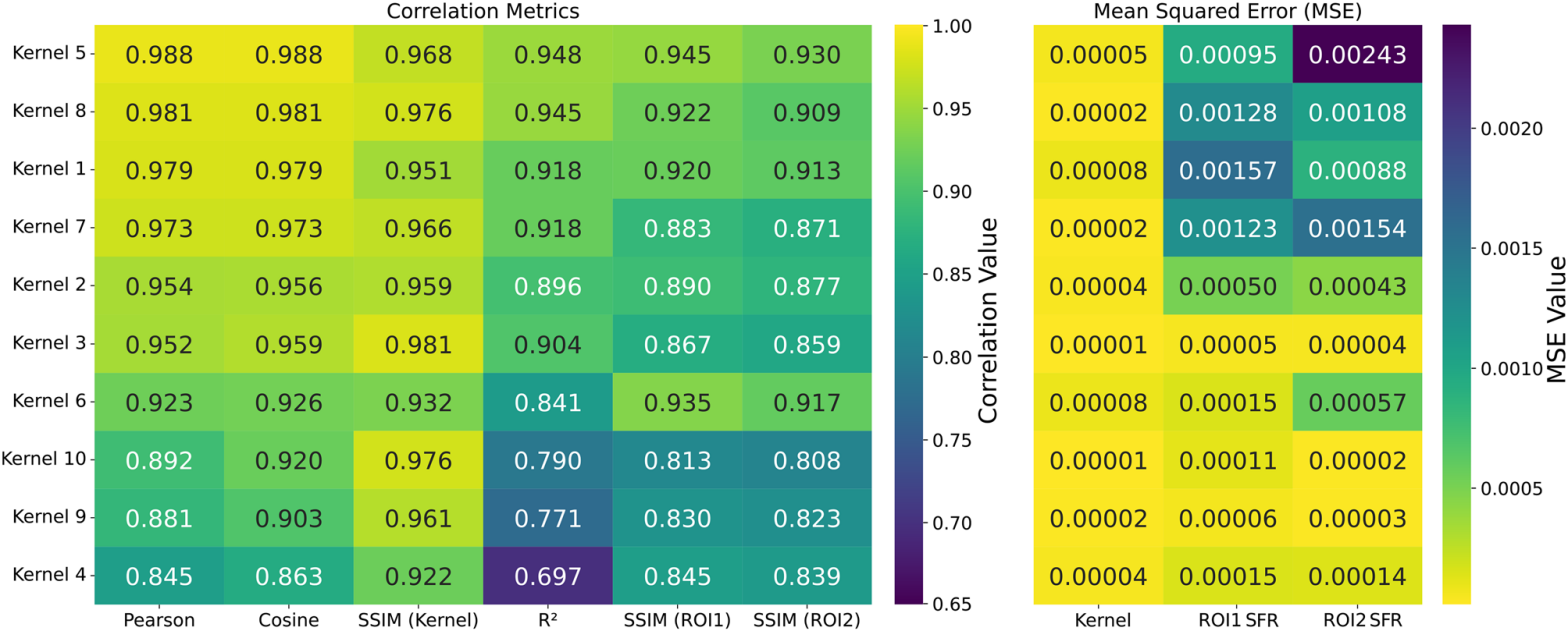
Heatmaps comparing the performance of ten estimated blur kernels against their true kernel using various metrics. The left heatmap shows correlation metrics (Pearson, Cosine, SSIM, $\textrm{R}^{2}$) between the estimated and true kernels, as well as the SSIM of the convolved ROIs. The right heatmap displays the MSE between the estimated and true kernels and the estimated ROIs. Higher values in the correlation map and lower values in the MSE map indicate better performance of the kernel estimation and image restoration.

The correlation metrics between the estimated and true kernels were summarized in Fig. 6. The heatmap was arranged from best to worst according to the Pearson correlation coefficient, and the corresponding statistics were reported across all ten defocus positions as shown in Table 2. Across all kernels, the Pearson correlation was $0.94 \pm 0.05$, the Cosine similarity was $0.94 \pm 0.04$, the coefficient of determination $R^{2}$ was $0.86 \pm 0.08$, and the SSIM for kernel comparison was $0.96 \pm 0.02$. The corresponding MSE between estimated and true kernels was $(3.88 \pm 2.51)\times 10^{-5}$, with minimum and maximum values bounded between $1.09 \times {10}^{-5}$ and $8.27\times{10}^{-5}$, respectively. These results indicate that, on average, the estimated kernels were consistent with the ground-truth kernels across all defocus levels.

**Fig. 7:**
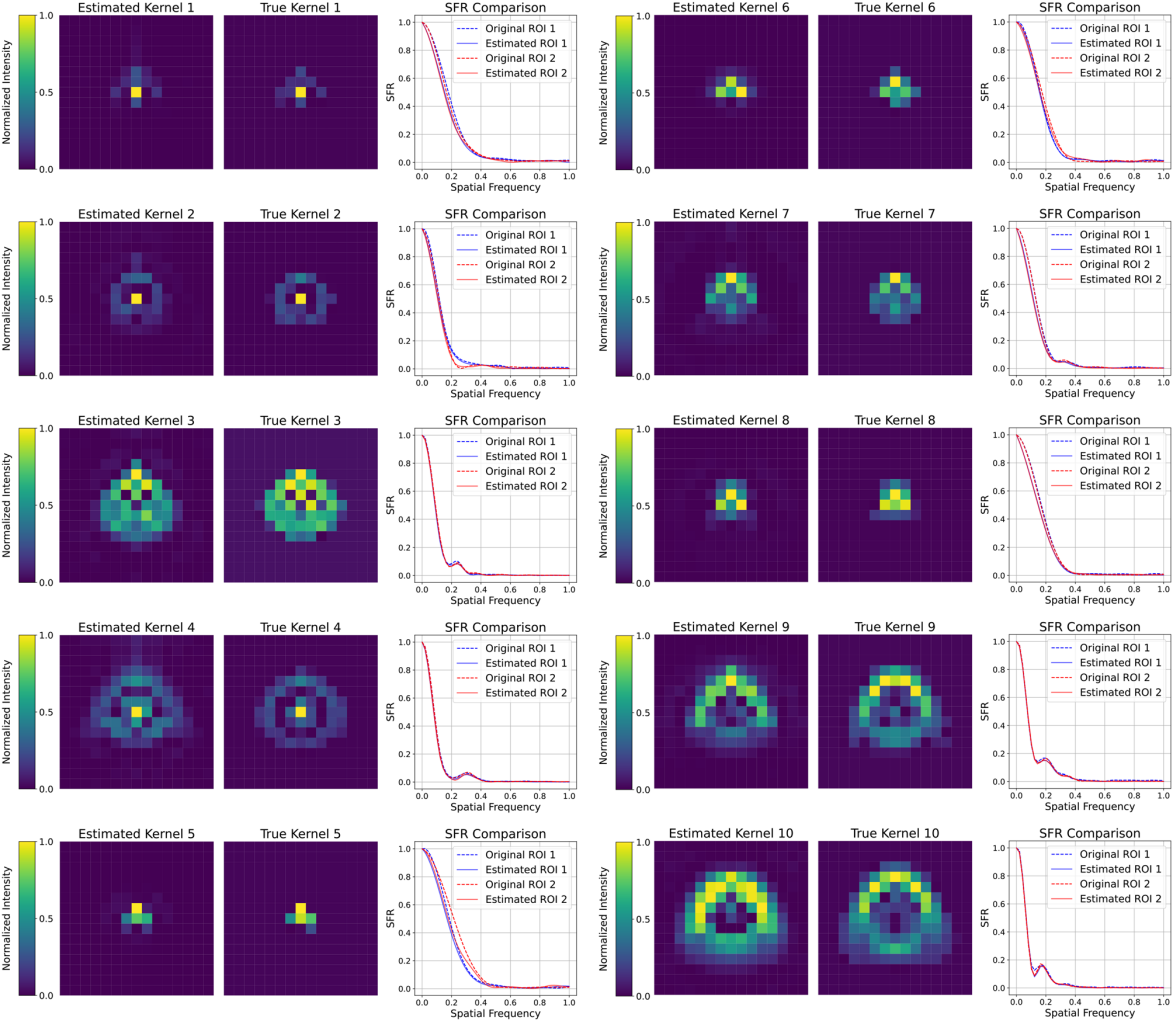
Comparison of ten estimated blur kernels with their corresponding true kernels. For each kernel pair, a plot shows the SFR comparison between the original ROIs and the ROI convolved using the estimated kernel. The visual similarity between the estimated and true kernels, along with the closeness of the restored ROI’s SFR to the original SFR, indicates the accuracy of the blur estimation.

Heatmaps showing true and estimated kernels for visual comparison are shown in Fig. 7. This plot also shows the mismatch between the true SFR (from the originally blurred image) and the estimated SFR (calculated from the convolved image). It can be seen that, for most test cases, the estimated kernels were visually similar to the corresponding true kernels. This was consistent with the SFR plots for both ROIs, which showed small differences and substantial overlap in many cases.

The ranking of individual kernels in Fig. 6 confirms this overall trend. Kernel 5 shows the best performance across multiple metrics, attaining Pearson and Cosine similarity values close to 0.99 and an $R^{2}$ score of approximately 0.95. Kernel 8 demonstrates comparable performance, while Kernel 3 exhibits the highest SSIM value for kernel comparison. Kernel 4 was the lowest-performing estimate, with an $R^{2}$ score of about 0.70 and a Pearson correlation of about 0.85, and Kernels 9 and 10 also show relatively lower performance with $R^{2}$ scores around 0.77 and 0.79, respectively. Quantitatively, this pattern reflects the fact that the best estimations were obtained for kernels with a smaller spatial spread (kernels 1, 5, and 8), corresponding to lower defocus, whereas the errors increase as the kernel becomes more extended (e.g. kernels 4, 9, and 10). A plausible explanation was that the variance of the kernel data increases with defocus and was not fully captured by the 35 principal components used in the PCA representation. However, as the estimated kernels always showed an SSIM score greater than 0.92 and the resulting SFRs still exhibited an MSE on the order of ${10}^{-4}$, which was good enough for this work.

**Table 2:** Kernel-wise comparison between the proposed PCA+DE method and three blind deconvolution baselines. Values are the mean ± standard deviation over the ten test kernels.

Method	MSE	SSIM	Pearson	Cosine	$R^{2}$
PCA (proposed)	$3.88\times 10^{-5} \pm 2.51\times 10^{-5}$	$0.959 \pm 0.018$	$0.937 \pm 0.047$	$0.945 \pm 0.039$	$0.863 \pm 0.079$
Pan2016	$2.60\times 10^{-4} \pm 2.62\times 10^{-4}$	$0.610 \pm 0.113$	$0.741 \pm 0.080$	$0.761 \pm 0.082$	$0.538 \pm 0.124$
Deblur-INR (BIRD)	$4.39\times 10^{-4} \pm 5.58\times 10^{-4}$	$0.572 \pm 0.232$	$0.632 \pm 0.251$	$0.654 \pm 0.252$	$0.350 \pm 0.376$
GKPILE	$4.79\times 10^{-4} \pm 6.71\times 10^{-4}$	$0.473 \pm 0.252$	$0.553 \pm 0.263$	$0.587 \pm 0.257$	$0.118 \pm 0.525$

**Fig. 8:**
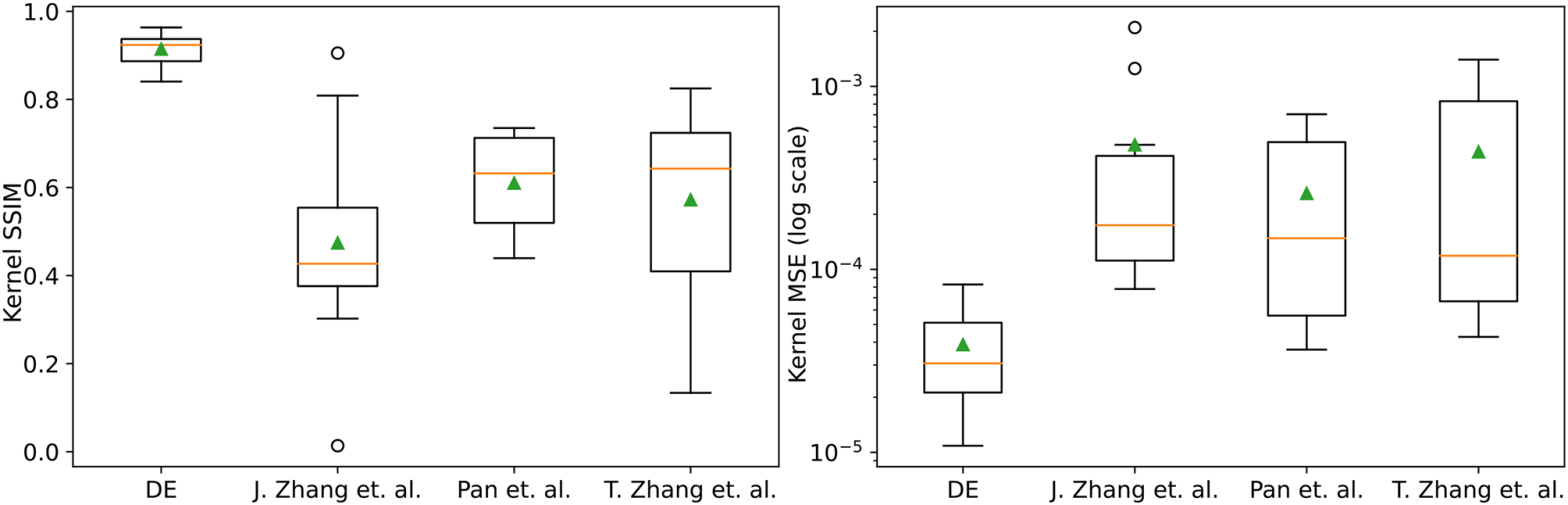
Box plot showing the SSIM and the MSE between the true kernel and the estimated kernel by the baseline methods compared to the method proposed in this work. It can be seen that the method proposed in this work has low MSE and high SSIM compared to the other methods. This could be because of the informed learning of the PCA model from the existing kernel database, which is not available to the other methods.

#### Comparison with existing Kernel-estimation methods

To further assess the kernel-estimation accuracy of the proposed PCA approach, it was compared against three recently published deblurring methods that explicitly estimate a blur kernel: the edge-based blind deconvolution algorithm of Pan et al.^21^, the implicit neural representation method Deblur-INR by T. Zhang et al.^22^, and the generative kernel prior model GKPILE by J. Zhang et al.^23^. All methods were evaluated on the ten traffic-sign images and corresponding 15$\times$15 ground-truth PSFs used in Fig. 6. For each method and defocus setting, the MSE, SSIM, Pearson correlation, cosine similarity, and coefficient of determination R^2^ were computed between the estimated kernel and the ground-truth kernel. The results are shown in Fig. 8 and the summary is given in Table 2. The figure (Supplementary Fig. S1) compares the estimated kernels to the true kernels from all the tested methods as heatmaps.

Across the ten kernels, the proposed PCA estimator with DE optimization achieved results better than the three state-of-the-art models. The results show that the proposed method consistently yielded kernels that were closer to the ground truth in both spatial and correlation-based metrics. It is important to note that the PCA estimation methods operate in a learned PCA manifold of PSFs derived from the same Zemax model, and the DE optimizer searches within this low-dimensional, physically plausible kernel space. In contrast, the three methods that were tested are always applied in a blind fashion to a single blurred image, without explicit prior knowledge of the underlying automotive lens design. The stronger performance of the PCA method on this dataset, therefore, demonstrates the benefit of exploiting such model-based prior information for kernel estimation in a fixed optical system, rather than indicating a universal superiority over generic blind deconvolution methods for arbitrary blur types.

**Fig. 9:**
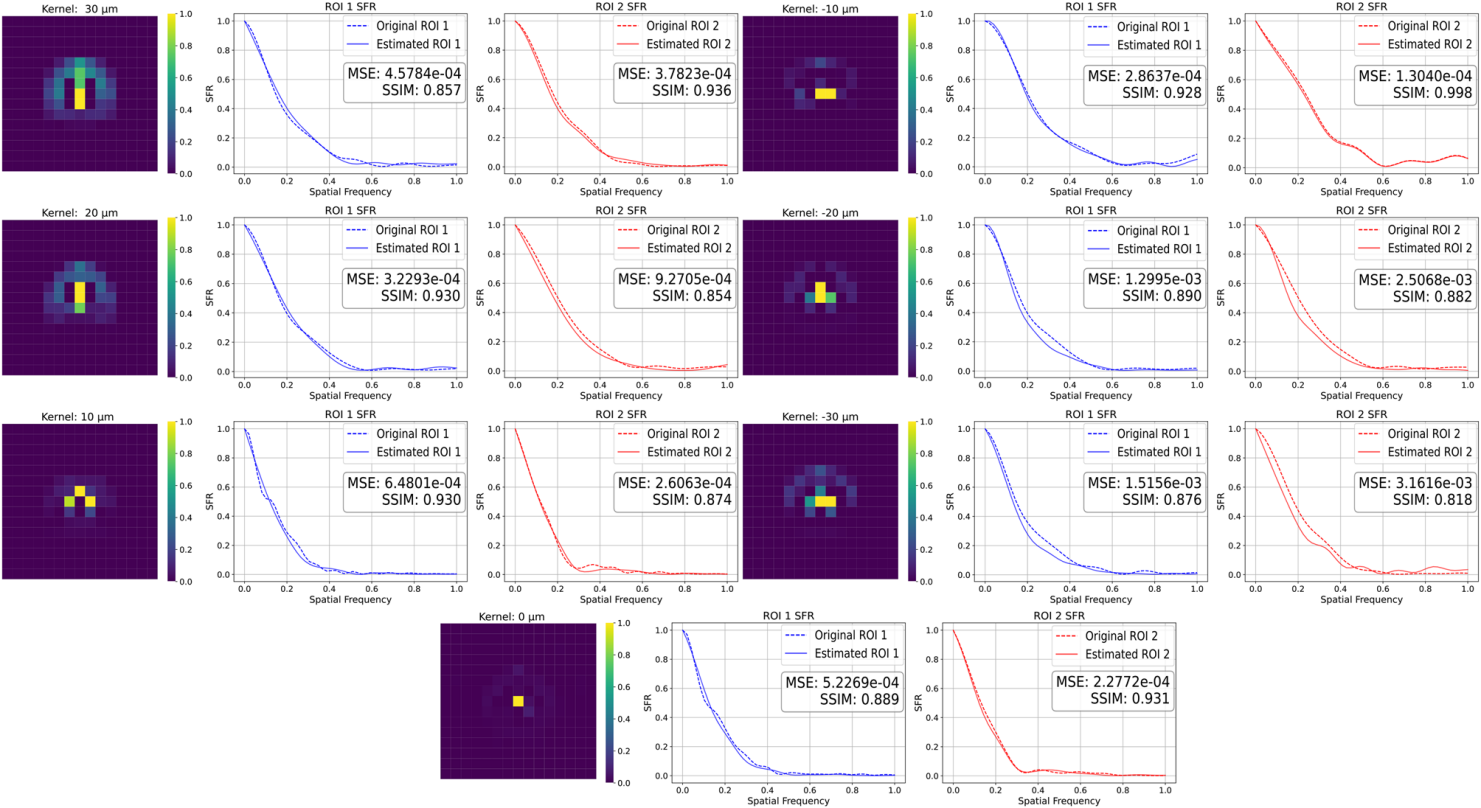
Estimated blur kernels for defocused images between −30 $\upmu \textrm{m}$ and +30 $\upmu \textrm{m}$. For each kernel, a plot shows the SFR comparison between the original ROIs and the ROI convolved using the estimated kernel. The visual similarity between the estimated and true kernels, along with the closeness of the restored ROI’s SFR to the original SFR, indicates the accuracy of the blur estimation. Also labeled in the SFR plots were the MSE and SSIM between the estimated convolved ROI and the original ROI.

### Kernel estimation on camera data

After the method was developed, the algorithm was validated using traffic sign images from a modified automotive camera. In this modified camera, the sensor was moved towards and away from the objective, inducing defocus in both directions (positive being away from the objective and negative being towards the objective). Seven such images (defocus between −30 and +30 $\upmu \textrm{m}$ at 10 $\upmu \textrm{m}$ intervals) were used for validating the algorithm, and the results of the kernel estimation and SFR matching were shown in Fig. 9. Since true kernels for the modified camera were not known, SFR matching and SSIM between the blurred and estimated ROIs will serve as an indication of goodness-of-fit of the kernel. It can be seen in Fig. 9 that the estimated SFR and the blurred SFR curves show similar values for different spatial frequencies, leading to a qualitatively good overlap between the two curves. The plots in Fig. 9 also show the MSE for each SFR. The maximum and minimum MSE were $3.2 \times {10}^{-3}$ and $1.30 \times {10}^{-4}$, respectively. Across all defocus settings and both ROIs, the mean SFR MSE was $(3.6 \pm 2.2)\times 10^{-4}$, while the corresponding SSIM was $0.90 \pm 0.05$. These values suggest that, for the tested scenes and defocus settings, the estimated kernels reproduce the measured SFR with reasonably small residual error. The figure also shows the SSIM between the estimated ROI and the blurred ROI, which was always higher than 0.82, indicating that the convolved reference ROI exhibits a similar edge structure to the original blurred ROI. This corroborates the results of the SFR match and shows that the method estimates kernels that could be plausible local kernel estimates for this camera and defocus. Since the current dataset provides suitable traffic sign ROIs only in the central part of the image, these results should be interpreted strictly as local validation. The spatial variation of kernels across the full field of view was not experimentally measured in this study. It should be noted that the noise in the SFR plots was not well captured by the model. This was due to the PCA model being trained on the kernels generated by Zemax, which does not account for sensor noise, leading to the model being unable to capture the noise and its impact on the kernel and the SFR.

### In-field state monitoring and image deconvolution

**Fig. 10:**
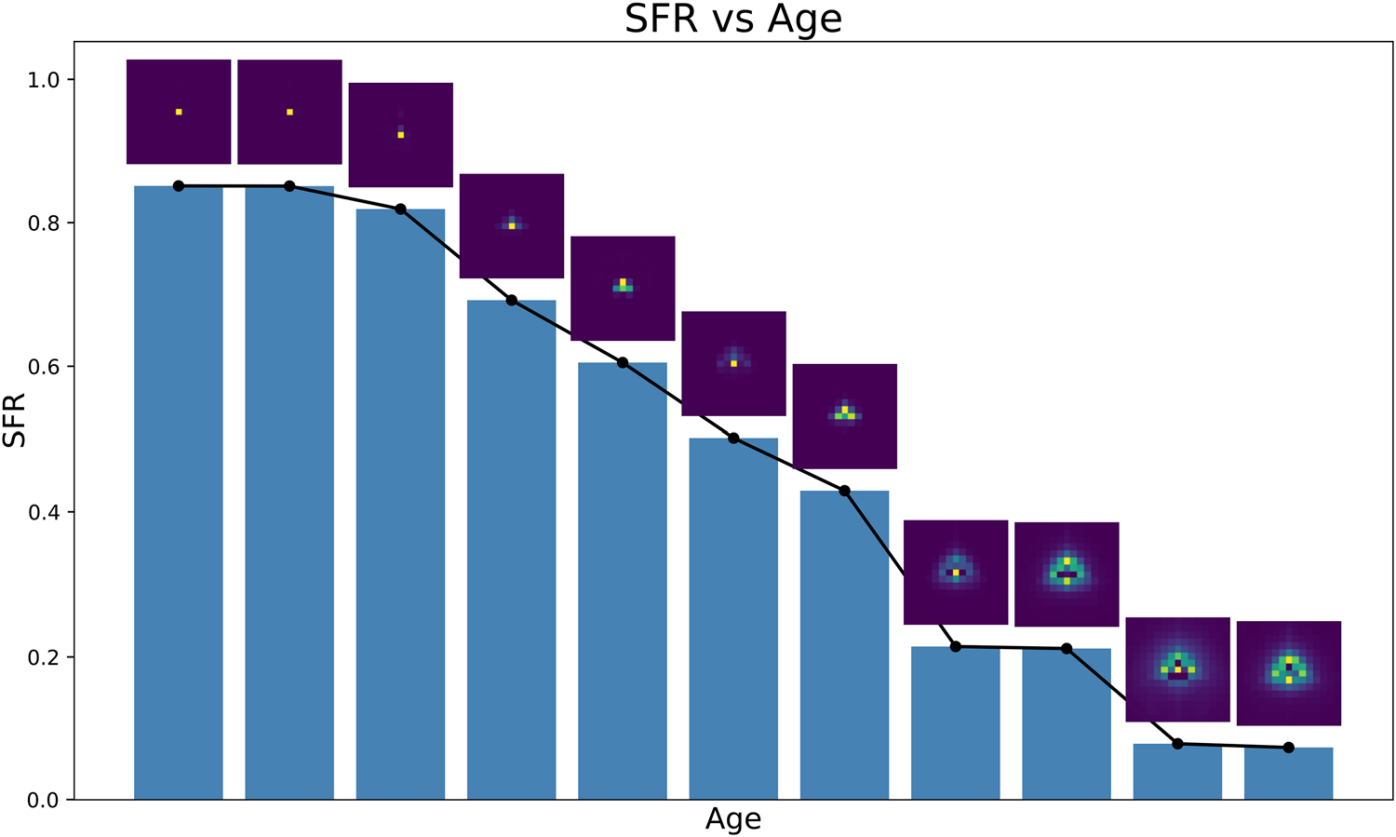
Plot showing the general behaviour of SFR and kernels with age. Bar heights represent SFR values at 0.25 cycles/pixel for different defocus levels (increasing from left to right). Above each bar, the corresponding estimated blur kernel is shown.

The kernel estimation method presented in this work can be applied as a first step in state monitoring of automotive cameras by tracking the changes in the estimated kernels and their SFR over time. One generalized depiction of the change in SFR and kernel with age is shown in Fig. 10. As seen in Sections "Kernel estimation on synthetic data" and "Kernel estimation on camera data", the algorithm can estimate changes in kernel from very low defocus (less spread, as in kernel 5 and kernel 6) to significant spread (kernel 3, 9, and 10). It should also be noted that the kernel estimation could also lead to a good estimation of the aberrations, such as astigmatism and coma, as shown in the work by Kalinkina et al.^48^. When the estimated kernels and hence the estimated PSFs were coupled with optical models from Zemax, they could help estimate the state of the optical elements in the objective (radii of surfaces, refractive indices, and the distance between elements). This “reverse path” of modeling will help in estimating the state of the camera using the image captured. The “forward path” of modeling is the technique generally defined in optical simulations where the state of the camera determines the quality of the image captured by the camera (e.g., image simulation in Zemax qualitatively analyzes the impact of a change in lens properties on the output image). Extending the approach beyond ROI-based kernel estimation to a full-field spatially variant PSF map does not necessarily require interpolating kernels across the image plane. Instead, the estimated local kernels can be treated as sparse measurements that constrain a calibrated forward optical model of the lens–sensor system (e.g., the Zemax model). In a practical setting, the inverse step can be formulated over a low-dimensional set of physically meaningful system-state parameters (e.g., effective defocus and dominant aberration or misalignment terms) that best reproduce the observed local PSFs at the measured field positions. Once this system state is estimated, the forward model can be evaluated on a dense field grid to generate a consistent full-image spatially variant PSF distribution across the sensor, enabling space-variant deconvolution and providing a physically interpretable link between observed blur and likely optical changes. By periodically estimating the kernel and comparing it to previous kernel estimates, it is possible to detect shifts in the optical performance of the system. This could be either a change in kernel size, a change in the SFR, or both. Tracking the SFR over time provides a quantitative measure of how the camera’s imaging performance is changing, offering insights into the rate of degradation. SFR tracking combined with an understanding of the minimal threshold needed to ensure that safety-critical features like object detection and collision avoidance function as intended would help in decision-making for maintenance or replacement. Although industry testing standards do not define a lower threshold for SFR or PSF before maintenance is needed, a commonly used industrial threshold for end-of-line validation is an SFR value of 0.5 at 60 line-pairs/mm. This threshold serves as a pass/fail criterion in many automotive camera testing protocols. Although SFR tracking alone could be a minimal viable state monitoring method, the SFR is a one-dimensional metric that leads to a binary state of the camera (good or bad). Coupling that with kernel estimation gives more insights into the state of the camera and its optical components. This is a non-invasive and low-effort technique for continuous monitoring of camera health, which facilitates early identification of aging effects before they compromise safety-critical features.

In addition to state monitoring, the estimated kernels can also be utilized for image deconvolution to restore image sharpness. Kernel estimation was performed over small, localized regions because they were spatially variant. These regions were selected around slanted edges present in various parts of the image, allowing multiple spatially localized kernels to be estimated. It may not be feasible to estimate kernels for the entire image for various reasons. These may include the positions of the traffic signs in the image, the number of pixels present on the slanted edge of the traffic sign, or even the absence of any slanted edge in the image. However, obtaining kernels from critical regions, especially those relevant to object detection, will enable targeted deblurring. This approach can improve image quality in areas that are important for machine vision algorithms.

## Conclusion and outlook

The presented algorithm demonstrates that the proposed PCA- and DE-based framework can estimate blur kernels that, on the synthetic dataset, achieve high correlation metrics and low MSE values across a range of defocus conditions. Although performance was best for kernels with smaller spreads, corresponding to less severe blur, the algorithm maintains statistically significant agreement with the reference kernels even for more extensively blurred cases. Validation on camera data indicates that, for the available scenes and ROIs, the method can reproduce SFR curves and edge structures with reasonably high similarity. However, these results should be interpreted as an initial proof of concept rather than a fully validated automotive diagnostic tool. While the results show correlation between estimated and real blur characteristics, the current method was explicitly validated as a localized, ROI-based estimator of blur kernels around well-defined slanted edges. The dependence of the method on slanted edges limits applicability to scenes without these features, which could be extended by using other edge-rich features, such as text regions on traffic signs, lane markings, or other high-contrast man-made features. However, in scenes where suitable edges are absent, kernel estimation is inherently limited, and alternative image quality assessment methods may be necessary. Blur in automotive cameras is spatially variant across the field of view, and a single estimated kernel cannot represent the full image. The method relies on extracting ROIs with sufficient edge contrast and known geometry, which restricts its applicability to scenes containing suitable traffic signs or edge structures. Moreover, kernel estimation was performed on small patches (45$\times$45 pixels), meaning the spatial coverage was limited unless extended with more ROIs across the image. As a result, while the method can be used as a first step towards state monitoring using specific targets, it cannot provide full-image deblurring without additional sampling or interpolation strategies. It is also important to note that the 45$\times$45 pixel patch size imposes a practical lower bound on the apparent size of the traffic sign in the image, limiting kernel estimation at longer distances due to insufficient edge resolution. Furthermore, occlusions or damage (e.g., dirt, scratches) on the lens or target area may degrade the reliability of edge detection and SFR extraction, impacting kernel accuracy. Additionally, sensor noise or compression artifacts may lead to poor SFR matching since the current PCA model does not account for them.

Future work will extend the in-field study to multiple traffic sign positions across the image plane. This will enable explicit mapping of spatially variant PSFs and evaluation of interpolation strategies from sparse kernel estimates to full-field PSF distribution. This work will also include noise mitigation strategies such as filtering, which is needed for real-life scenarios as seen in the camera validation results. An extension of this work will also focus on enhancing the algorithm’s performance with a larger number of diverse kernels, which could involve increasing the number of principal components while also exploring alternative dimensionality reduction techniques. In the next steps, the authors plan to directly estimate a PSF from the model, which could lead to a more accurate estimation of the higher-order aberrations and thus the state of the optical system. The successful estimation of blur kernels would open more possibilities for applications in state monitoring and image deconvolution, with the potential to significantly improve image quality and facilitate more accurate analysis.

## Supplementary Information

Supplementary Information.

## Data Availability

The datasets used in the current study are available from the corresponding author on reasonable request.
