# Supplementary figures and images for: Quantitative Kernel estimation from traffic signs using slanted edge spatial frequency response as a sharpness metric
Source: Sci Rep. 2026 Feb 19;16:7387. doi: 10.1038/s41598-026-40556-w (PMC12923804; doi:10.1038/s41598-026-40556-w)

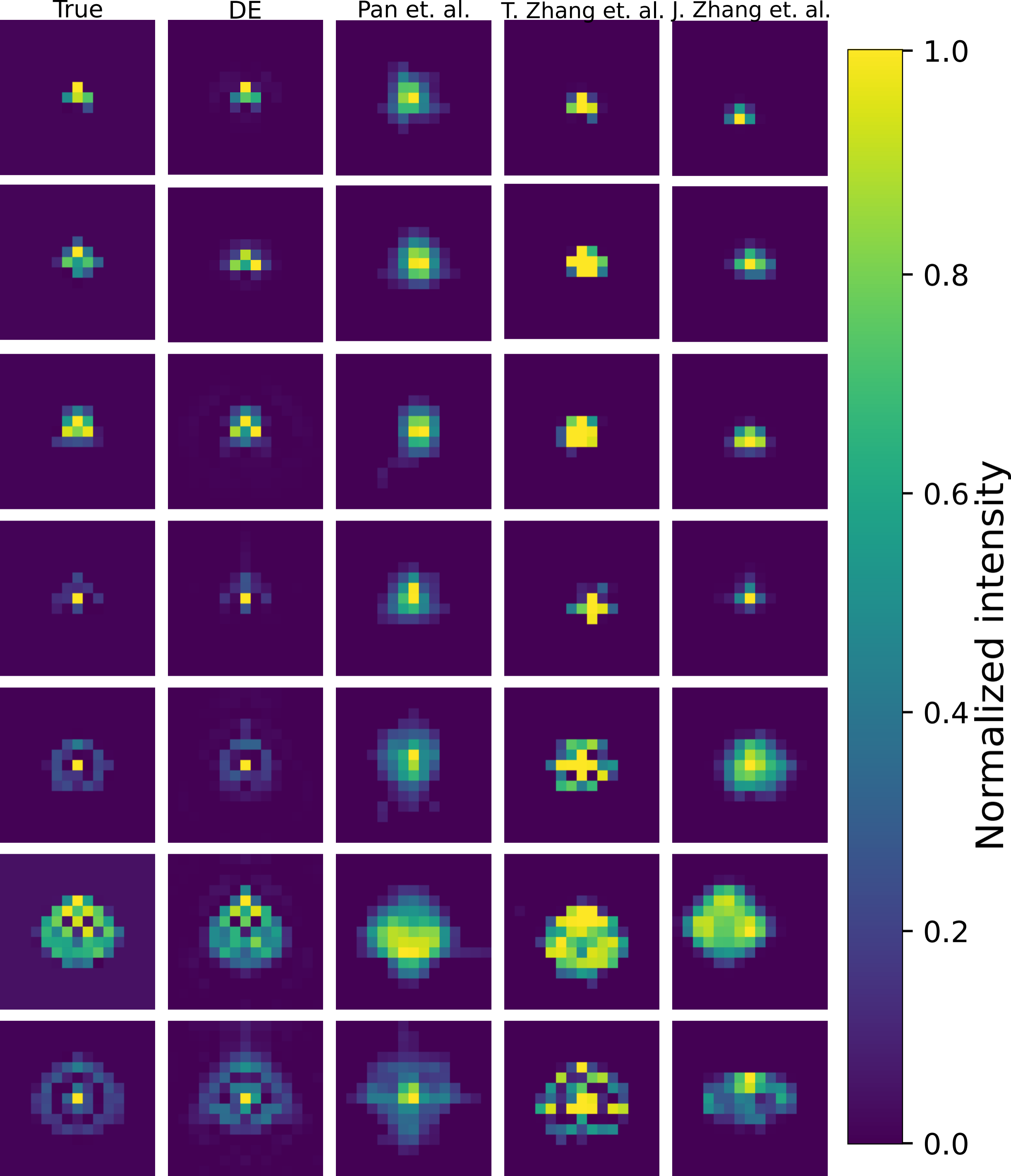

Supplement: Supplementary file 1 — Supplementary Information. [file 41598_2026_40556_MOESM1_ESM.pdf]
